# Time domain double slit interference of electron produced by XUV synchrotron radiation

**DOI:** 10.1038/s41598-023-33039-9

**Published:** 2023-04-15

**Authors:** T. Kaneyasu, Y. Hikosaka, S. Wada, M. Fujimoto, H. Ota, H. Iwayama, M. Katoh

**Affiliations:** 1 SAGA Light Source, Tosu, 841-0005 Japan; 2 Institute for Molecular Science, Okazaki, 444-8585 Japan; 3 Institute of Liberal Arts and Sciences, University of Toyama, Toyama, 930-0194 Japan; 4 Graduate School of Advanced Science and Engineering, Hiroshima University, Higashi-Hiroshima, 739-8526 Japan; 5 Synchrotron Radiation Research Center, Nagoya University, Nagoya, 464-8603 Japan; 6 Sokendai (The Graduate University for Advanced Studies), Okazaki, 444-8585 Japan; 7 Hiroshima Synchrotron Radiation Center, Hiroshima University, Higashi-Hiroshima, 739-0046 Japan

**Keywords:** Atomic and molecular physics, Quantum physics, X-rays

## Abstract

We present a new realization of the time-domain double-slit experiment with photoelectrons, demonstrating that spontaneous radiation from a bunch of relativistic electrons can be used to control the quantum interference of single-particles. The double-slit arrangement is realized by a pair of light wave packets with attosecond-controlled spacing, which is naturally included in the spontaneous radiation from two undulators in series. Photoelectrons emitted from helium atoms are observed in the energy-domain under the condition of detecting them one by one, and the stochastic buildup of the quantum interference pattern on a detector plane is recorded.

## Introduction

Wave-particle duality is one of the most fundamental concepts in quantum mechanics. The concept has previously been beautifully demonstrated by the double-slit experiment, in which particles such as electrons^1,2^, atoms^3,4^, molecules^5–7^ and neutrons^8^ passing through the double slit exhibit interference patterns in the intensity distribution on a detection screen, similar to those obtained with light^9,10^. The interference pattern reflects the probability density given by the modulus squared of the particle’s wavefunction. The essence of wave-particle duality is revealed in these experiments; while the particle behaves as a delocalized wave as it propagates, it is always detected as a well-localized particle that stochastically hits the screen, forming an interference pattern that becomes visible with increasing number of detected particles. Indeed, one-by-one detection of the particles allows the recording of the emergence of the interference pattern under single-particle conditions; that is, there is at most one particle in the interferometer at any time. The real-time observation of this buildup process with single-electrons^2^ is widely known as the “most beautiful experiment in physics^11^”, demonstrating the electron’s interference with itself. Furthermore, the recent advances in nanofabrication technology allows one to observe the buildup of quantum interference pattern even for heavy dye molecules^12^, paving the way to explore the boundary between quantum and classical physics.

Since there is an analogy between spatial diffraction and temporal dispersion in optics^13^, the observation of double-slit interference with particles is not restricted to conventional space-domain experiments but can be extended to time-domain experiments with matter wave packets that propagate in vacuum. To date, time-domain double-slit interference with particles has been observed for a slow atomic beam chopped by an atomic mirror^14^, and photoelectron wave packets produced by a pair of femtosecond laser pulses^15,16^. In these experiments, the time-separated wave packets temporally spread in free space during propagation and overlap each other, leading to the appearance of an interference pattern in the spatio-temporal distribution. Moreover, photoelectron interference has been observed using extremely short temporal slits in the attosecond regime prepared by the field ionization of atoms interacting with few-cycle laser pulses^17^. The double-slit interference of photoelectron wave packets has also been theoretically studied for multiple attosecond slits^18^, and for the effect of the carrier-envelope phase^19^. Recently, vortex-shaped photoelectron wave packets resulting from interference have been observed using a pair of cross-circularly polarized femtosecond laser pulses^20^. In the meantime, two-color coherent pulses generated by seeded FELs enabled control of photoelectron angular distribution through photoelectron interference^21^.

In spite of these progress in time-domain interference experiments with photoelectrons, the recording of the buildup process of the interference pattern for single-electrons has not been achieved, although this approach is of particular importance for interpreting quantum mechanics as demonstrated in the space-domain experiments. This is presumably due to experimental difficulties in observing the particle’s position on a screen in time-domain experiments, and in ensuring that there is only a single-electron in the interferometer at any time. Further, since a coherent pulse pair is essential for preparing the double-slit arrangement in the time-domain, previous studies used laser pulses at optical wavelengths. While extending the photoelectron interference experiments to shorter wavelengths is highly desirable for probing ultrafast processes in matter, wavelengths shorter than the vacuum ultraviolet have been inaccessible to double-slit experiments so far, due to the difficulty of generating a coherence pulse pair.

This paper reports a new time-domain double-slit experiment in the extreme ultraviolet (XUV) and attosecond regime using synchrotron radiation, and demonstrates a recording of the buildup of the quantum interference pattern for single-photoelectrons. Unlike the previously reported studies, we realized the time-domain double-slit arrangement in the XUV wavelength region using spontaneous radiation emitted from a bunch of relativistic electrons in a synchrotron ring. Although synchrotron radiation has poor temporal coherence in general, we use the usually hidden ability of undulator radiation which enables the use of mutual longitudinal coherence between time-separated light wave packets^22–26^. By controlling the motion of a relativistic electron in a synchrotron ring, we can adjust the spacing between the two light wave packets at the attosecond level^27^, which works as a temporal double-slit. Taking advantage of the high repetition rate of synchrotron radiation (i.e. a small number of photons per pulse), we can easily suppress the photoionization yield per pulse to ensure that there is only a single-photoelectron in the interferometer at any given time. The interfering photoelectron wave packets emitted from helium atoms are observed in the energy-domain with a one-by-one detection scheme, allowing us to visualize the buildup of the quantum interference pattern of single-photoelectrons which stochastically arrive at the detector plane.

## Results and discussion

### Time-domain double-slit by synchrotron radiation

**Figure 1:**
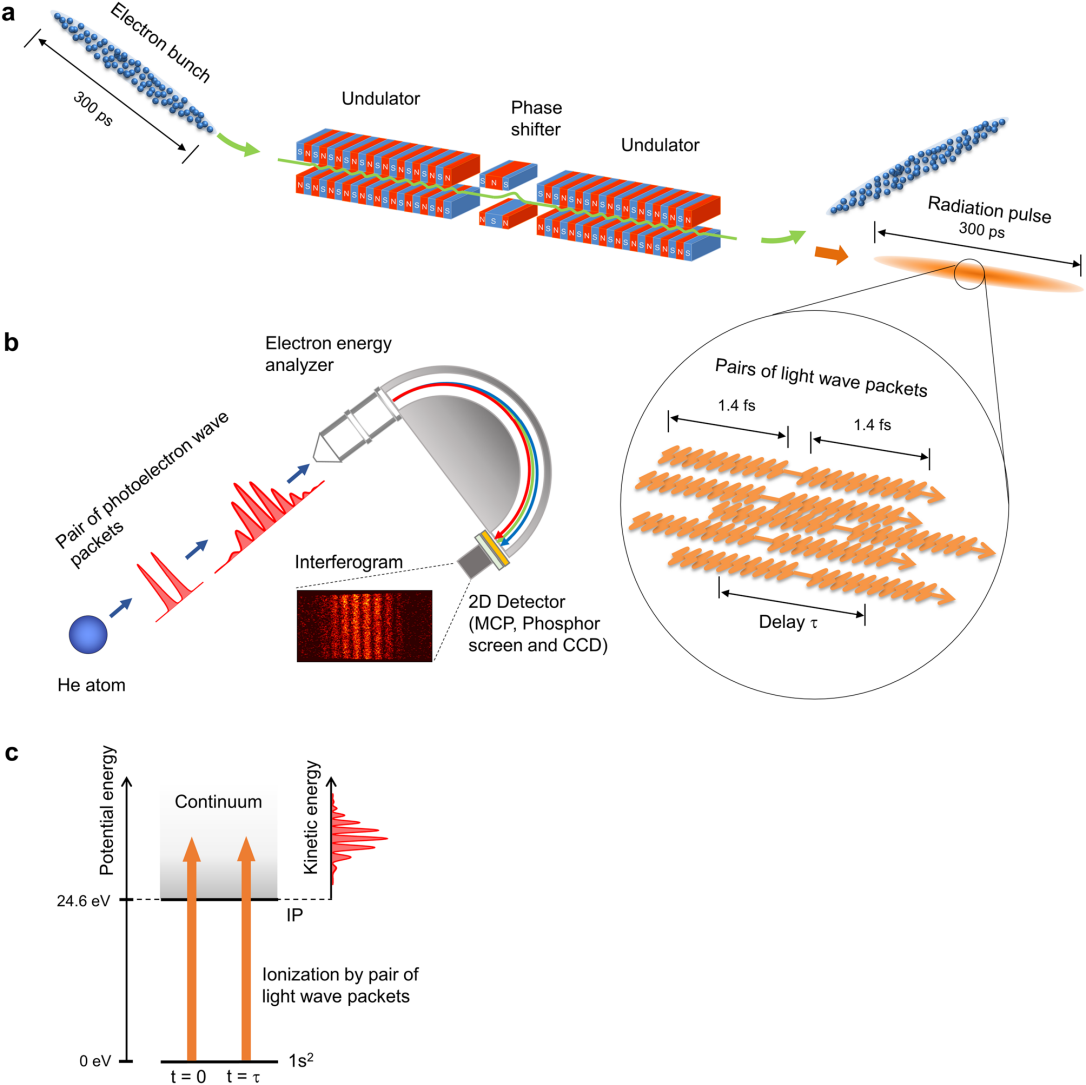
Experimental layout for time-domain double-slit interference by synchrotron radiation. (**a**) Spontaneous radiation is emitted from a bunch of relativistic electrons passing through the tandem-undulator in the UVSOR-III synchrotron ring. Each of the relativistic electrons in the bunch emits a pair of light wave packets, reflecting the sequential undulating motion in two undulators. Numerous pairs of light wave packets are randomly distributed in the radiation pulse. (**b**) A pair of photoelectron wave packets is emitted from a helium atom that interacts with the pair of light wave packets. The interfering photoelectron wave packets are observed as an interferogram on the detection plane of the hemispherical electron energy analyzer. The buildup of quantum interference pattern is monitored by a 2D detector which allows for one-by-one detection of the photoelectrons, which stochastically arrive at the detector plane. (**c**) Energy level diagram of helium atom and the ionization process by a pair of light wave packets.

Figure 1 shows the experimental layout. To produce the temporal double-slit, we use a tandem-undulator system in which each relativistic electron in the bunch emits a pair of light wave packets that has a mutual coherence between them. The waveform of each light wave packet is characterized by 10-cycle oscillations with a rectangular envelope, reflecting the undulating motion of the relativistic electron in the undulator^27^. The radiation wavelength is set to about 43 nm. The duration of the 10-cycle oscillations is approximately 1.4 fs, which defines the width of the temporal slit. The time delay between the light wave packets in the pair defines the temporal separation between the two slits, and is precisely tuned in the femtosecond regime with attosecond resolution using a phase shifter magnet located between the two undulators.

A pair of light wave packets sequentially interacts with a helium atom, producing a pair of photoelectron wave packets that propagate in free space (Fig. 1b). Due to the momentum dispersion of the matter waves, the first and second components of the photoelectron wave packet pair temporally spread in free space and overlap each other, leading to the appearance of the interference pattern in coordinate space^15,16^. This wave packet interference gives rise to the appearance of interference fringes in energy or momentum distributions. Therefore, the measurement of the photoelectron spectrum makes the observation of wave packet interference experimentally possible. Figure 1c shows a schematic drawing of the energy level of helium atom and the ionization process using a pair of light wave packets. Note that in the actual experiment, an incoherent mixture of numerous pairs of light wave packets in the radiation pulse interacts with a helium atom, leading to the production of pairs of photoelectron wave packets that are randomly distributed in a temporal duration of 300 ps. However, the incoherent properties among the photoelectron wave packets related to different light wave packets are canceled out in the observable quantities [see Methods]. Consequently, the interference pattern observed in this study directly reflects the quantum interference between photoelectron wave packets produced by a pair of light wave packets.

In order to visualize the buildup of the interference pattern, we observe the wave packet interference in the energy-domain. We record photoelectron images using a two-dimensional (2D) position-sensitive detector mounted in a hemispherical electron energy analyzer as shown in Fig. 1b. The 2D detector consists of a micro-channel plate (MCP), a phosphor screen, and a charge-coupled device (CCD) camera. We adjust the light intensity to ensure that there is only a single photoelectron in the electron analyzer at any given time, enabling the observation of photoelectron interference. The buildup process of the quantum interference pattern is monitored by accumulating the CCD images.

### Buildup of photoelectron interference

**Figure 2:**
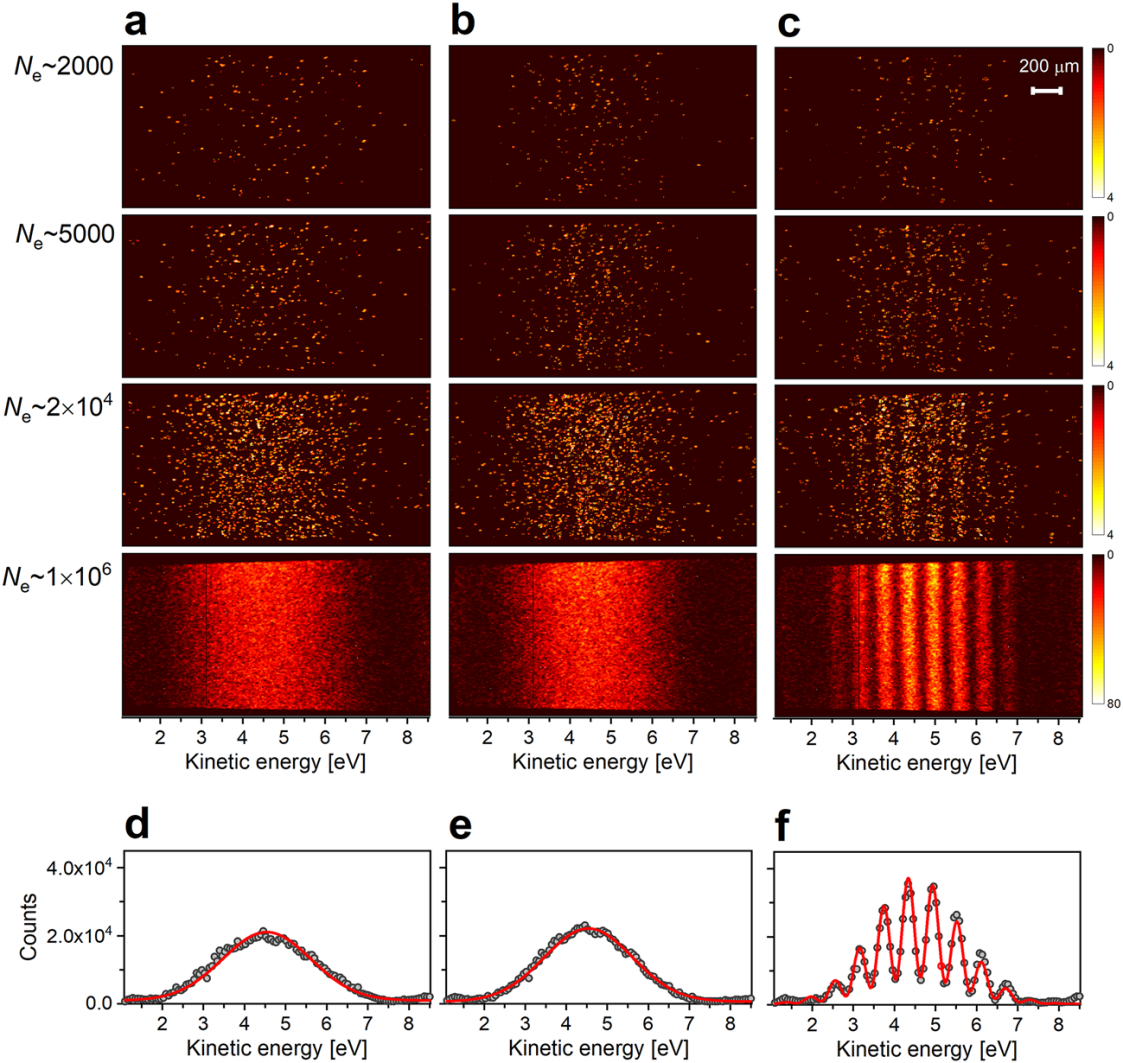
One-by-one detection of single-photoelectrons. (**a**–**c**) CCD images of photoelectrons observed for radiation from (**a**) the upstream undulator, (**b**) the downstream undulator, and (**c**) the tandem-undulator. The phase shifter current was kept constant during the measurements. The total number of detected electrons are shown at the left side of the panels (**a**). The color bar ranges from 0 to 4 electrons in the upper three panels, and from 0 to 80 electrons in the images obtained at 1 × 10^6^ electrons. (**d**–**f**) One-dimensional distributions obtained by projecting the electron intensities in the images of 1 × 10^6^ electrons onto the energy axis. The interference fringes were fitted by a model curve using Eq. (3).

Figure 2 compares the CCD images of photoelectrons recorded with the radiation from single- (Fig. 2a,b) and tandem-undulator configurations (Fig. 2c). The single-undulator configuration is prepared by setting the pole gap of one of the two undulators to its maximum, where the radiation power of the undulator becomes negligibly small. The field strength of the phase shifter magnet was fixed with the same coil current during the measurement. While the horizontal axis of the image shows the kinetic energy of the photoelectron, the vertical axis represents the ionization points of atoms along the light propagation axis in the gas cell. When the single-undulator is used, the helium atom is ionized by light wave packets emitted from either the upstream or downstream undulator, leading to the production of single (not-paired) photoelectron wave packets. Therefore, no interference pattern can be exhibited on the CCD image. In this case, the photoelectron distribution on the detector plane simply reflects the spectral distribution of the undulator radiation, which is characterized by a 10% width (FWHM) of the central photon energy. With increasing numbers of photoelectrons, broad structures centered at around 4.5 eV become visible in panels of Fig. 2a,b. On the other hand, interfering photoelectron wave packets are produced when the radiation from the tandem-undulator is used to ionize helium atoms. The CCD images in Fig. 2c shows the two-dimensional distribution of photoelectrons measured with pairs of light wave packets. The CCD image after accumulation of 2000 electrons shows an almost random distribution. An interference pattern exhibiting the wave-like nature of single photoelectrons appears at 2 × 10^4^ electrons, and becomes clearer as the number of electrons is increased.

One-dimensional photoelectron spectra are obtained from the two-dimensional images at *N*_*e*_ ~ 1 × 10^6^ by integrating the photoelectron counts onto the horizontal axis (Fig. 2d–f). The photoelectron spectra obtained by the single-undulator show single peak structures centered at around 4.5 eV kinetic energy. In contrast, the photoelectron spectrum measured with the tandem-undulator is characterized by an interference pattern. The photoelectron spectra in Fig. 2d,e can be well fitted by a single Gauss function which approximately represents the spectral distribution of the radiation from the single-undulator. The interference pattern in Fig. 2f, on the other hand, can be fitted by the probability density for pairs of photoelectron wave packets in the energy-domain (see “Methods”). In the fitting, the width of the temporal slit determines the envelope of the interference pattern which is experimentally obtained by the fitting for the photoelectron spectra measured with the single-undulator. The free parameters in the fitting were the time delay between the light wave packets and the fringe contrast. The theoretical curve well fits the interference pattern, and time delays are obtained to be 6.9 fs. The contrast of the interference fringes is limited to 0.7, mainly due to the reduction in temporal coherence resulting from electron beam properties such as the energy spread and angular divergence, which smear the time delay^23^.

### Control of double-slit separation

**Figure 3:**
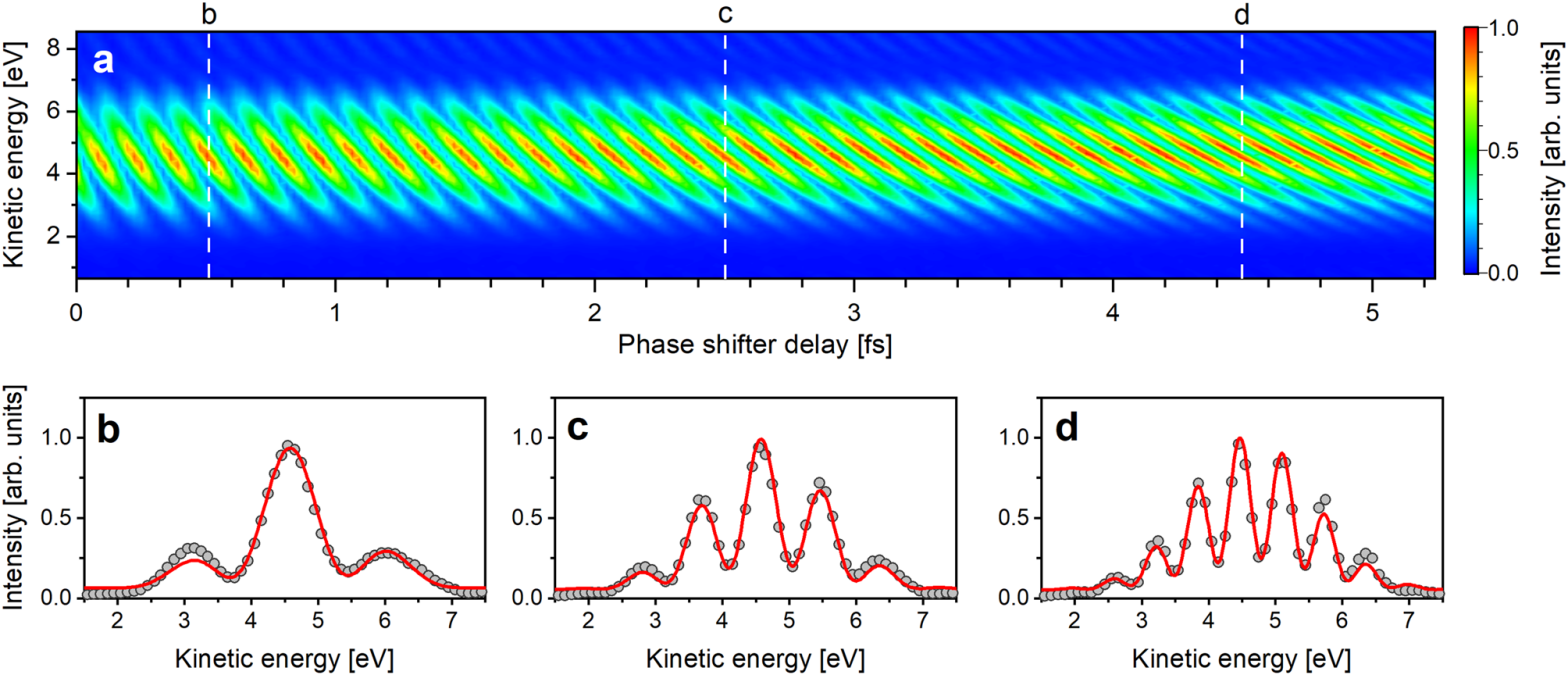
Time-domain photoelectron interferogram. (**a**) The photoelectron interferogram consists of photoelectron spectra measured as a function of the phase shifter delay. (**b**–**d**) Photoelectron spectra obtained at phase shifter delays of 0.51, 2.50, and 4.52 fs (vertical dashed lines in **a**). The photoelectron spectra are fitted by Eq. (3). The experimental data points and fitting curves are shown by gray circles and red curve, respectively, in (**b**–**d**).

Figure 3a shows the time-domain interferogram, which consists of photoelectron spectra measured as a function of time delay. The horizontal axis shows the time delay produced by the phase shifter magnet. To obtain the absolute time delay τ, it is required to add a minimum delay of approximately 1.9 fs which corresponds to the sum of the temporal duration of the light wave packet and the time delay due to the slippage effect in the drift space between the two undulators. The vertical axis is the kinetic energy of the photoelectron. The interferogram exhibits a periodical modulation of photoelectron intensity on the two-dimensional plot. Similar to the discussion on the interference between the electron wave packets in the bound state^22^, the periodic intensity modulation can be explained by time-domain Ramsey interference between the photoelectron wave packets. When the time delay is varied, constructive and destructive interference occurs between the plane waves which constitute the photoelectron wave packets. The interference condition is determined by the phase difference *ωτ*. Here, the photon frequency is given by *ω* = (*E* + *E*_IP_)/*ħ* where *E* and *E*_IP_ are the kinetic energy of photoelectron and ionization potential of the atom, respectively^15^. Therefore, the photoelectron intensity oscillates at a temporal period of around 140 as. The time-domain Ramsey interference is clearly visible over the whole area of the interferogram, indicating that the photoelectron wave packet interference can be precisely controlled by adjusting the temporal separation between the two light wave packets at the attosecond level.

Figures 3b–d show the photoelectron spectra at phase shifter delays of 0.5, 2.5, and 4.5 fs (indicated by vertical dashed lines in a), showing interference patterns dependent on the time delay. The photoelectron spectra in Fig. 3 are well fitted by Eq. (3), and the time delays are obtained to be 2.41, 4.40, and 6.41 fs, respectively. The photoelectron spectra exhibit fringe structures that vary with the time delay. This observation is a manifestation of the fact that the temporal separation between the two slits determines the spacing between the interference fringes, as in the space-domain double-slit interference. Similar to double-slit interference in the space-domain, the number of interference fringes increases with increasing time delay. This can be qualitatively explained by considering the interference condition between plane waves with specific momenta which constitute the photoelectron wave packets. Constructive or destructive interference occurs between the plane wave components when the phase difference is an even or odd multiple of π. As the time delay becomes longer, two neighboring kinetic energies that satisfy the constructive interference condition become closer. Therefore, in Fig. 3b–d, as the time delay increases, the spacing between fringes becomes narrower, and thus the number of fringes increases.

## Conclusion

We have observed the buildup of quantum interference patterns for time-separated photoelectron wave packets emitted from helium atoms under the single-photoelectron condition. The results manifest the applicability of synchrotron light sources to conduct the time-domain double-slit experiment, demonstrating the wave-particle duality of single-photoelectrons emitted from atoms. The time-domain interference presented in this work relies on the use of longitudinal coherence within light wave packet pairs emitted from individual relativistic electrons in the bunch. The photoelectron distribution at the detector plane reflects the probability density in the energy-domain, revealing interference patterns which depend on the temporal separation between the two slits.

Unlike previous experiments which used ultrashort laser pulses, we employed a synchrotron light source to prepare the time-domain double-slit in the XUV and attosecond regime. This method, therefore, can be extended to much shorter wavelengths with powerful tunability of wavelength, polarization, and cycle number in the light wave packet. Extending the photoelectron interference experiment to short wavelengths is highly desirable since it offers the possibility to study ultrafast electron motion following core-ionization in atoms and molecules as well as condensed matter, taking advantage of experiments involving core-electrons such as element selectivity^28^. However, such interference experiments have not yet been realized owing to the difficulty of producing time-separated double pulses with well-defined waveforms at short wavelengths. In addition, we believe that this method could be used to study the decoherence effect of a sample interacting with its environment. In particular, a wide range of sample environments can become a target in the x-ray region owing to its high penetrating power.

## Methods

### Tandem-undulator system

The experiments were performed at the light source development beamline BL1U of the UVSOR-III synchrotron^29^. The tandem-undulator system consists of two identical APPLE-II undulators with a phase shifter magnet between them. Both undulators have a period length and number of periods of 88 mm and 10, respectively. The two undulators were set to provide fundamental radiation at a wavelength of 43 nm with horizontally linear polarization. As a result of the sequential undulating motion in the tandem-undulator system, each electron in the bunch emits a pair of 10-cycle light wave packets. The beam current in the synchrotron ring was about 5 mA during the measurement, which corresponds to a few 10^8^ electrons in the 300-ps long electron bunch. Therefore, a few 10^8^ light wave packets are randomly distributed in the 300-ps radiation pulse. The duration of the light wave packet was approximately 1.4 fs, which corresponds to the coherence time of the radiation pulse from a single-undulator. The time delay between the light wave packet pair is naturally longer than the coherence time of the radiation pulse from the single-undulator. We tuned the time delay by using the phase shifter magnet, which has a three-pole wiggler configuration. The time delay was calibrated with respect to the coil current of the phase shifter magnet by measuring the time-domain Ramsey fringes on the 1s6p resonance of the helium atom^22^.

### Single-electron detection

The light beam from the undulator consists of 300-ps radiation pulses with a 90 MHz repetition rate. The central part of the light beams was selected by a 0.4-mm diameter pinhole located 9 m downstream from the midpoint of the two undulators. The light beam passing through the pinhole was focused by a toroidal mirror. The electron energy analyzer (MBS, A1) was placed at the focal point of the light beam. The photoelectrons were observed at a fixed angle of 55 degrees with respect to the polarization plane. The arrival position of the photoelectrons on the detector plane was recorded by using a CCD camera. (Sony, XC-ST30).

The UVSOR synchrotron provides radiation pulses at a 90 MHz repetition rate. The photon flux, ionization cross sections, gas pressure and length of the interaction region are 1 × 10^12^ photons/s, 5 Mb^30^, 2 × 10^–1^ Pa and 5 mm, respectively. Under the experimental condition, the rate of photoionization event is about 1 × 10^8^/s and one photoelectron is generated every radiation pulse on average. The acceptance angle of the electron analyzer is about 0.4% of the full solid angle and the transmission efficiency of the entrance slit of the analyzer is 20%. Consequently, the rate of photoelectrons entering the analyzer is estimated to be 8 × 10^4^ electrons/s in the present study. Assuming 60% detection efficiency of the MCP detector, there is a close agreement between estimation and experiment on the electron counting rate. The estimated and experimental counting rates are 5 × 10^4^ and 6 × 10^4^ electrons/s, respectively. The overall detection efficiency for a photoelectron emitted from a helium atom is estimated to be 5 × 10^–4^. In addition, the flight time of the photoelectron from the entrance of the analyzer to the detector is about 250 ns. These conditions ensure that there is only a single-photoelectron in the electron analyzer at any given time, allowing for the observation of single-photoelectron interference.

### Interference between photoelectron wave packets

We follow the procedure described by Wollenhaupt et al.^15,16^. The photoelectron wave packet is represented by a superposition of plane waves

1 $$\begin{array}{c}\psi \left(x,t\right)=\frac{1}{\sqrt{2\pi }}{\int }_{0}^{\infty }dEc\left(E\right){e}^{i\left({k}_{e}x-Et/\hslash\right)},\end{array}$$

where $E$, *c*(*E*), *k*_e_ are the kinetic energy, probability amplitude, and wave vector of the photoelectron, respectively. For photoionization by a pair of light wave packets with time delay τ, the probability amplitude is given by

2 $$\begin{array}{c}c\left(E\right)\propto \left(1+{e}^{i\omega \tau }\right)\widetilde{E}\left(\omega \right),\end{array}$$

where ω and $\widetilde{E}\left(\omega \right)$ are the frequency and Fourier component of the single light wave packet. The photon frequency ω is given by (*E* + *E*_IP_)/*ħ*. Assuming that the ionization cross section is constant within the spectral width of the ionizing pulse, the photoelectron spectrum is given by

3 $$\begin{array}{c}{\left|c\left(E\right)\right|}^{2}\propto 2\left(1+\mathrm{cos}\omega \tau \right){\left|\widetilde{E}\left(\omega \right)\right|}^{2}.\end{array}$$

The photoelectron spectrum is modulated due to the interference between the photoelectron wave packets. While the shape of the envelope determined by the single pulse spectrum is unchanged, the interference fringe structure strongly depends on the time delay.

When the atom interacts with *N* pairs of light wave packets, the probability amplitude is approximately given by

4 $$\begin{array}{c}c\left(E\right)\propto \sum_{j}\left(1+{e}^{i\omega \tau }\right){e}^{i\omega {\delta }_{j}}\widetilde{E}\left(\omega \right),\end{array}$$

where δ_j_ is time delay of *j*th light wave packets which is measured with respect to the first one^20^. Therefore, the photoelectron spectrum is obtained by

5 $$\begin{aligned} \left| {c\left( E \right)} \right|^{2} & \propto \mathop \sum \limits_{j,k} \left| {1 + e^{i\omega \tau } } \right|^{2} e^{{i\omega \left( {\delta_{j} - \delta_{k} } \right)}} \left| {\tilde{E}\left( \omega \right)} \right|^{2} \\ & = \mathop \sum \limits_{{j = 1 \left( {j = k} \right)}}^{N} \left| {1 + e^{i\omega \tau } } \right|^{2} e^{{i\omega \left( {\delta_{j} - \delta_{k} } \right)}} \left| {\tilde{E}\left( \omega \right)} \right|^{2} + \mathop \sum \limits_{{j,k = 1 \left( {j \ne k} \right)}}^{N} \left| {1 + e^{i\omega \tau } } \right|^{2} e^{{i\omega \left( {\delta_{j} - \delta_{k} } \right)}} \left| {\tilde{E}\left( \omega \right)} \right|^{2} \\ & = 2N\left( {1 + \cos \omega \tau } \right)\left| {\tilde{E}\left( \omega \right)} \right|^{2} \\ \end{aligned}$$

Similar to photoionization by single double-pulse, the photoelectron spectrum shows an interference pattern determined by the time delay. The only difference between the two cases is the transition probability, which is proportional to the number of the light wave packets—in other words, the light intensity. It is noted that the incoherence property of the synchrotron radiation originating from the randomly distributed electrons in the bunch is canceled out in the above expression due to the random phase relationship between the light wave packets emitted from different electrons. This indicates that it is possible to observe the interference between photoelectron wave packets even with the use of spontaneous synchrotron radiation from a bunch of relativistic electrons when the tandem-undulator is utilized as a light source.

To observe the photoelectron interference by synchrotron radiation, it is essential to produce light wave packets which have identical waveforms. While the energy spread and angular divergence of the electron beam reduce the waveform identity of individual light wave packets^23^, the effect from the electron beam on the waveform shapes is sufficiently small at the XUV wavelengths for the electron beam of the UVSOR-III synchrotron, as demonstrated in the interferometric measurements^22,23,27^.

## Data Availability

The datasets used and/or analyzed during the current study are available from the corresponding author on reasonable request.
